# Reply: Tuberculosis screening in migrants to the EU/EEA and UK

**DOI:** 10.1183/13993003.01535-2023

**Published:** 2023-11-02

**Authors:** Dominik Zenner, Frank Cobelens, Ibrahim Abubakar

**Affiliations:** 1 Faculty of Population Health Sciences, University College London, London, UK; 2 Wolfson Institute of Population Health, Queen Mary University of London, London, UK; 3 Amsterdam University Medical Centers, location University of Amsterdam, Department of Global Health, Amsterdam, The Netherlands; 4 Amsterdam Public Health, Global Health, Amsterdam, The Netherlands

## Abstract

We would like to thank N. Köhler and co-workers for their correspondence regarding our recent paper [1], comparing and contrasting it with their large pan-European study. Their study collected aggregate country-specific tuberculosis (TB) incidence rates as measured by infectious disease surveillance systems in the country of arrival (CoA) [2] and compared these to World Health Organization (WHO) TB incidence estimates from their respective country of origin (CoO). The authors found considerable differences between these incidence rates and conclude that there are many factors, other than incidence in the CoO, which determine TB risk. The authors therefore call for more granular screening policies which consider a wider range of factors including country-specific incidence as measured in CoAs.

## Tweetable abstract

**Tuberculosis incidence estimates from countries of origin alone are often insufficient to predict TB prevalence among migrants** https://bit.ly/3PDc35g

*Reply to N. Köhler and co-workers*:

We would like to thank N. Köhler and co-workers for their correspondence regarding our recent paper [1], comparing and contrasting it with their large pan-European study. Their study collected aggregate country-specific tuberculosis (TB) incidence rates as measured by infectious disease surveillance systems in the country of arrival (CoA) [2] and compared these to World Health Organization (WHO) TB incidence estimates from their respective country of origin (CoO). The authors found considerable differences between these incidence rates and conclude that there are many factors, other than incidence in the CoO, which determine TB risk. The authors therefore call for more granular screening policies which consider a wider range of factors including country-specific incidence as measured in CoAs.

We entirely agree with their conclusion that TB incidence estimates from CoOs alone are often insufficient to predict TB prevalence among migrants. WHO TB incidence estimates have several well-described limitations and are only a good proxy in countries with minimal under- or over-reporting in contexts with highly performing surveillance systems and excellent access to and quality of health services, or where a national prevalence survey has recently been conducted [3]. Better estimates can be gained after inventory studies, including capture–recapture, but these are utilised only for few countries, many of them not CoOs. Screening policies should consider several other factors, including the nature of migrants’ journeys and arrival, biological susceptibility and time since arrival; some national screening programmes already do [4, 5]. We also agree that it is likely that country-specific rates, as measured in CoAs, can be a significant addition to inform screening decisions if they are available and robust.

TB prevalence among migrants is also dependent on individual risk factors, including socioeconomic or circumstantial factors, often related to living conditions in the CoO, their mode of departure and hazards in transit and on arrival, and of course confounders related to their health, including comorbidities. These impact directly, and also indirectly, for example through decreasing access to care. Among these, the likely significant, but hard to measure TB risk *en route* is an important explanatory factor for the observed risk differences between the CoO and CoA, and might explain at least part of the excess TB risk among asylum seekers compared with other migrant typologies, and is emphasised in both papers [1].

It is worth noting that the direct comparison of our study with that of Vasiliu *et al.* [2] is limited by the studies’ scope, design and type of underlying data. Vasiliu *et al.* [2] used aggregate country-level data from the entire European Union/European Economic Area/UK region. Aggregate country-level data provides a powerful overview but is limited in its ability to adjust for confounding, and its validity depends on the quality and homogeneity of underlying country data.

Our study [1] benefits from pooling programmatic individual-level screening data from four European countries, including data from more than 2 million individuals. While only including four countries with large screening programmes, our analysis is the first to quantify the extent of confounding and interaction effects of different risk factors. We replicated Vasiliu *et al.* [2]'s CoO/CoA comparison for estimated TB prevalence rates using our individual-level data, and also found considerable variation between CoO/CoA pairs; however, prior to presenting such data, detailed work should be undertaken to allow comparability with WHO data and careful adjustments for confounding at individual and programme levels, which are all possible with our dataset, but outside the scope of this correspondence.

In conclusion, our different but synergistic papers both fill an important gap in the literature. Much more remains to be done, including further analysis of our screening dataset to continue to build a robust evidence base for screening policy makers in the future.

## Shareable PDF

10.1183/13993003.01535-2023.Shareable1 This one-page PDF can be shared freely online.
Shareable PDF ERJ-01535-2023.Shareable
