# Supplementary material for: Reply: Tuberculosis screening in migrants to the EU/EEA and UK
Source: Eur Respir J. 2023 Nov 2;62(5):2301535. doi: 10.1183/13993003.01535-2023 (PMC10620474; doi:10.1183/13993003.01535-2023)
Supplement: Supplementary file 1 [file ERJ-01535-2023.Shareable.pdf]

EUROPEAN RESPIRATORY JOURNAL
CORRESPONDENCE
D. ZENNER ET AL.

SHAREABLE PDF

Reply: Tuberculosis screening in migrants to the EU/EEA and UK

Dominik Zenner[1,2,3,4], Frank Cobelens[3,4] and Ibrahim Abubakar [1]

[1]Faculty of Population Health Sciences, University College London, London, UK. [2]Wolfson Institute of Population Health, Queen Mary University of London, London, UK. [3]Amsterdam University Medical Centers, location University of Amsterdam, Department of Global Health, Amsterdam, The Netherlands. [4]Amsterdam Public Health, Global Health, Amsterdam, The Netherlands.

Corresponding author: Dominik Zenner (d.zenner@qmul.ac.uk)

Shareable abstract (@ERSpublications)
Tuberculosis incidence estimates from countries of origin alone are often insufficient to predict TB prevalence among migrants https://bit.ly/3PDc35g

Cite this article as: Zenner D, Cobelens F, Abubakar I. Reply: Tuberculosis screening in migrants to the EU/EEA and UK. *Eur Respir J* 2023; 62: 2301535 [DOI: 10.1183/13993003.01535-2023].

This extracted version can be shared freely online.

Copyright ©The authors 2023.

This version is distributed under the terms of the Creative Commons Attribution Licence 4.0.

Received: 18 Sept 2023
Accepted: 20 Sept 2023

Reply to N. Köhler and co-workers:

We would like to thank N. Köhler and co-workers for their correspondence regarding our recent paper [1], comparing and contrasting it with their large pan-European study. Their study collected aggregate country-specific tuberculosis (TB) incidence rates as measured by infectious disease surveillance systems in the country of arrival (CoA) [2] and compared these to World Health Organization (WHO) TB incidence estimates from their respective country of origin (CoO). The authors found considerable differences between these incidence rates and conclude that there are many factors, other than incidence in the CoO, which determine TB risk. The authors therefore call for more granular screening policies which consider a wider range of factors including country-specific incidence as measured in CoAs.
